# One or Many Recoveries? Recoveries in the Plural for a Better Understanding of One's Healing Journey

**DOI:** 10.1111/hex.70209

**Published:** 2025-03-15

**Authors:** Elena Faccio, Michele Rocelli, Lia Bitetti, Giuseppe Salamina, Susanna Brunelli, Federica Mangione, Ludovica Aquili

**Affiliations:** ^1^ Department of Philosophy, Sociology, Education and Applied Psychology (FISPPA) University of Padua Padua Italy; ^2^ Psychologist at the community for substance abusers called Ca' delle Ore Breganze Italy; ^3^ Ministry Delegate for Health, Department of Mental Health Local Health Unit of Turin Turin Italy; ^4^ Peer Support Worker, or Expert by Experience, independent activist in Peer Support Verona Italy; ^5^ Peer Support Worker, or Expert by Experience, Diapsi Vercelli, Volunteer Association Vercelli Italy

**Keywords:** clinical recovery, mental health, personal recovery, recovery, relational recovery, theoretical framework of recovery

## Abstract

**Background:**

Since the 60s, the recovery‐oriented approach has greatly influenced mental health policy and practice, and much research has been devoted to exploring it. In the face of a generic definition of the ‘recovery’ construct, to which many articles refer, a closer examination of the literature reveals a plurality of theories and ways about how changes related to the recovery occur and how to evaluate them.

**Aims:**

This narrative review explores the different definitions of recovery available in the literature, by investigating the adjectives that qualify it and the theoretical construct the adjective refers to.

**Method:**

From the online databases PubMed, Scopus, Google Scholar and PsycINFO, 43 articles were selected for the review.

**Results:**

Seven definitions of recovery emerged, each supported by specific theoretical perspectives: clinical, personal, narrative, social, family, cultural and relational recovery. The adjectives refer to theoretical frameworks often very distant from each other and in epistemological competition; nevertheless, many papers assume a reconcilability and possible integration. The authors critically discuss the advantages and risks of considering such different constructs as complementary.

**Conclusions:**

Keeping theoretical descriptions and models of healing open and plural means enabling mental health practitioners not to monologise discourses of change by imposing their point of view on users. It means supporting users to authentically seek their healing pathways without conforming to clinicians' expectations. It also means abandoning misleading and naive simplifications and strictly using the appropriate terms relevant to the specific healing construct that researchers refer to from time to time. This is particularly important when it comes to the relational component, which seems to be receiving more and more attention in the literature, and about which there is more confusion.

**Patient or Public Involvement:**

The study involved two experts by experience, or peer support specialists, in a more than active role as components of the research team. They participated equally with the other team members in all phases of the work: the design and conduct of the study, the discussion of findings and advice about implications and dissemination.

## 1 Introduction

Many publications have dealt with the topic of recovery in mental health since the 1960s, with a surge in the last 20 years. However, there is still no clear and agreed definition of the construct. Its origins are in the medical model, oriented on the search for organic causes of symptoms. Anthony's definition (from 1993) is still considered the most accepted and shared: recovery is ‘a deeply personal, unique process of changing one's attitudes, values, feelings, goals, skills, and roles’ and ‘a way of living a satisfying, hopeful, and contributing life even within the limitations caused by illness’ (p. 527). ‘Recovery involves the development of new meaning and purpose in one's life as one grows beyond the catastrophic effects of mental illness’ ([1], p. 527) and is conceived as a cure, aimed at the remission of symptoms [2]. Despite its widespread use, much research points out the difficulty of applying this definition in today's clinical practice [3].

The term recovery has been variously interpreted in literature: it has been used to mean an approach, a model, a philosophy, a paradigm, a movement, a vision and, sceptically, a myth ([4], p. 38). An in‐depth analysis of the literature may show a lack of consensus regarding the definition of the construct ([5], p. 17). ‘The term appears to have a simple and self‐evident meaning, but the notion of recovery has become the focus of a considerable amount of confusion and debate among various groups in the mental health community’ ([6], p. 480). It is a contested concept among service users, caregivers, professionals and policymakers [7]. This ‘heterogeneity of perspectives’ reflects the interpretative complexity of mental distress and the coexistence of different cognitive paradigms. By using the same term (‘recovery’), one thinks of saying the same, but is this really the case? The coexistence of different perspectives on recovery may result from several factors: since the 1970s, two different meanings of the term have evolved in parallel [8].

The first, more conceptual, has been proposed by the World Health Organization and is based on longitudinal studies with a biomedical approach. The second derives from the movement of former patients, survivors, consumers or users of mental health services, which claimed the right of people with severe mental illness to live a life beyond the role of ‘psychiatric patient’ [9, 10]. Despite the recovery‐oriented movement's interest in the subjective nature of mental problems [11], the biomedical model and its emphasis on aetiology remain highly influential worldwide [12, 13].

These two perspectives, with their values and languages, are still present; sometimes their definitions are in strong tension, and sometimes they intertwine, generating insuperable ambiguities [2, 4]. Service users know that the assessment of their recovery status is carried out by service workers, and even if they disagree with the biomedical model, they use it to fulfil the criteria necessary to decide whether or not they are ready for discharge from the service. These are passepartout narratives, relevant to clinicians' expectations, which prove useful and are somewhat internalised [14]. The meanings of the recovery construct are, therefore, strongly influenced by the staff's view and recognition of the importance of medication use for future well‐being. It is impossible to separate the view of the user from that of the practitioner, regardless of the adequacy of the medical model, to represent the user's pathway and needs [14]. Anyway, the introduction of the dual perspective (Davidson, 2007), which summarises the technical knowledge with the expression ‘recovery from’ in opposition to the experiential knowledge, ‘to be in recovery’, is the basis of the subsequent enlargement of the meanings to be attributed to the construct. It represents the beginning of the expansion of viewpoints.

Given the typically Western tendency to medicalise the expression of mental diversity and to impose it, even for the current approach to services, it is essential to know and manage other recovery models available. Indeed, one must consider how, in different cultures, there are wide variations in how mental disorders are defined, interpreted, diagnosed and treated [15, 16].

In Western countries, the implementation of other recovery models, beyond the purely clinical and health aspects, has shifted the emphasis to the importance of aftercare, employment, social inclusion, education and quality of life [17, 18]. Furthermore, it has fostered a participatory, strengths‐based approach that values connectivity, hope, identity, meaning and empowerment [13, 19]. However, much more needs to be done so that other models, in addition to the more traditional clinical model, can represent both an intervention paradigm [3] and a service management model [20].

This article is part of the debate concerning the coexistence of different definitions of recovery and attempts to trace through a narrative review the origins of such confusion, as well as the plurality of interpretative lenses available to us, starting with an analysis of the adjectives by which the construct of recovery is qualified in the literature.

In this review, we decided to adopt a broad definition of ‘recovery’, considering it as a process that includes identity changes capable of representing both the mental health and substance use domains. In Italy, the distinction between these fields is blurred, as it has become necessary to integrate services into a unitary framework, both from a structural and financial point of view [21]. Although we do not fully agree with this simplification and reduction of complexity, in this paper we preferred to keep a broader look at the construct than possible.

## 2 Methods

The narrative overview turned out to be the most suitable method of filtering and analysing articles for our purpose. This method is useful for presenting a broad perspective on a topic and often describes the history or development of a problem or its management [22]. In this case, it offers a comprehensive narrative synthesis of previously published information on the various constructs defining recovery available in the literature that can serve to provoke reflection and controversy. Aware of the fact that usually the number of sources used to find the literature is incomplete, perhaps creating an insignificant knowledge base from which to draw a conclusion, we began by using a narrative and conceptual/thematic search with Google Scholar. This allowed us to identify the adjectives associated in the literature with the word ‘recovery’.

Eight adjectives were thus identified:

1. Clinical recovery

2. Personal recovery

3. Narrative recovery

4. Social recovery

5. Family recovery

6. Relational recovery

7. Cultural diversity in recovery

We then proceeded with a more systematic search using advanced research in the various databases (PubMed, Scopus, Google Scholar and PsycINFO, see Table 1) by setting in the title/abstract/keyword “clinical recovery” AND “Mental Health” and so on for all the adjectives considered. Due to the scarcity of articles found with ‘narrative recovery’, to increase the pool of articles to be analysed, we separated the adjective from the noun ‘recovery’ by entering in the search “narrative” AND “recovery” AND “mental health”. For the same reason, for ‘cultural recovery’ we separated “recovery” AND “cultural diversity”.

**Table 1:** Process of review.

Date of search	Databases	Years searched	Strings of terms in the field ‘title/abstract/keywords’	Records identified through database search	Selected articles after duplicate removal	Articles added after reading the references of selected articles or articles citing them	Selected articles
31.8.2024	PubMed (title/abstract): Scopus (article title/abstract/keywords) PsycINFO (abstract) Google Scholar (in title)	2005–2024	“Clinical Recovery” AND “Mental health”	PubMed:83 Scopus: 150 PsycINFO: 71 Scholar: 12	1	/	1
31.8.2024	PubMed (title/abstract): Scopus (article title/abstract/keywords) PsycINFO (abstract) Google Scholar (in title)	2005–2024	“Personal Recovery” AND “Mental health”	PubMed: 427 Scopus: 614 PsycINFO: 365 Scholar: 95	6	4	10
31.8.2024	PubMed (title/abstract): Scopus (article title/abstract/keywords) PsycINFO (abstract) Google Scholar (in title)	2005–2024	“Social Recovery” AND “Mental health”	PubMed: 55 Scopus: 101 PsycINFO: 55 Scholar: 26	4	8	12
31.8.2024	PubMed (title/abstract): Scopus (article title/abstract/keywords) PsycINFO (abstract) Google Scholar (in title)	2005–2024	“Family Recovery” AND “Mental health”	PubMed: 11 Scopus: 26 PsycINFO: 14 Scholar: 14	3	2	5
1.9.2024	PubMed (title/abstract): Scopus (article title/abstract/keywords) PsycINFO (abstract) Google Scholar (in title)	2005–2024	“Relational Recovery” AND “Mental health”	PubMed:4 Scopus: 9 PsycINFO: 5 Scholar: 7	2	3	5
1.9.2024	PubMed (title/abstract): Scopus (article title/abstract/keywords) PsycINFO (abstract) Google Scholar (in title)	2005–2024	“Narrative” AND “Recovery” AND “Mental health”	PubMed: 374 Scopus: 3 PsycINFO: 489 Scholar: 63	7	/	7
2.9.2024	PubMed (title/abstract): Scopus (article title/abstract/keywords) PsycINFO (abstract) Google Scholar (in title)	2005–2024	“Cultural diversity” AND “Recovery” AND “Mental health”	PubMed: 2 Scopus: 20 PsycINFO: 7 Scholar: 0	2	1	3

The years we considered range from 2005 to 2024. Based on a careful reading/thematic analysis of the identified abstracts, 43 articles were deemed suitable for narrative review as they were able to explore or define the construct (Table 2). Our focus was the selection of articles that accurately presented one of the seven adjectives associated with recovery. A narrative‐only, qualitative search would have excluded several articles that emerged from the more systematic search (see Table 2). Similarly, the systematic search also selected articles that, although they included the recovery adjective of interest in the abstract or title, did not go into it adequately. In some cases, it was necessary to supplement the search with articles that had been cited as references in other articles, although these were not filtered out by the systematic search. We followed in this process the indications offered by Green et al. [22]: clearly defining the topic to be summarised and explored through narrative review; keeping track of the databases, the terms and strings used, the period in which the search was conducted, and the criteria for inclusion and exclusion of articles.

**Table 2:** Selected articles.

Clinical recovery	(1) Pelletier, J. F., Davidson, L., Giguère, C. É., Franck, N., Bordet, J., and Rowe, M. (2020). Convergent and Concurrent Validity Between Clinical Recovery and Personal‐Civic Recovery in Mental Health. *Journal of Personalized Medicine*, 10(4), 163.
Personal recovery	(1) Leamy, M., Bird, V., Le Boutillier, C., Williams, J., and Slade, M. (2011). Conceptual Framework for Personal Recovery in Mental Health: Systematic Review and Narrative Synthesis. *The British Journal of Psychiatry*, 199(6), 445–452. (2) De Ruysscher, C., Vandevelde, S., Vanderplasschen, W., De Maeyer, J., and Vanheule, S. (2017). The Concept of Recovery as Experienced by Persons With Dual Diagnosis: A Systematic Review of Qualitative Research From a First‐Person Perspective. *Journal of Dual Diagnosis*, 13(4), 264–279. (3) Ness, O., Borg, M., Semb, R., and Karlsson, B. (2014). “Walking Alongside”: Collaborative Practices in Mental Health and Substance Use Care. *International Journal of Mental Health Systems*, 8, 1–8. (4) Davidson, L., and Roe, D. (2007). Recovery From Versus Recovery in Serious Mental Illness: One Strategy for Lessening Confusion Plaguing Recovery. *Journal of Mental Health*, 16(4), 459–470. (5) Stuart, S. R., Tansey, L., and Quayle, E. (2017). What We Talk About When We Talk About Recovery: A Systematic Review and Best‐Fit Framework Synthesis of Qualitative Literature. *Journal of Mental Health*, 26(3), 291–304. (6) Basso, L., Boggian, I., Carozza, P., Lamonaca, D., and Svettini, A. (2016). Recovery in Italy: An Update. *International Journal of Mental Health*, 45(1), 71–88. (7) Le Boutillier, C., Chevalier, A., Lawrence, V., Leamy, M., Bird, V. J., Macpherson, R.,… and Slade, M. (2015). Staff Understanding of Recovery‐Orientated Mental Health Practice: A Systematic Review and Narrative Synthesis. *Implementation Science*, 10, 1–14. (8) Brasier, C., Brophy, L., and Harvey, C. (2023). Constructing Recovery: A Lived Experience and Post‐Structuralist Exploration of How the Meaning of Personal Recovery and Rehabilitation Has Changed Over Time. *Australasian Psychiatry*, 31(5), 607–609. (9) Van Weeghel, J., van Zelst, C., Boertien, D., and Hasson‐Ohayon, I. (2019). Conceptualizations, Assessments, and Implications of Personal Recovery in Mental Illness: A Scoping Review of Systematic Reviews and Meta‐Analyses. *Psychiatric Rehabilitation Journal*, 42(2), 169. (10) Swords, C., and Houston, S. (2024). Service User Perspectives on Recovery: The Construction of Unfulfilled Promises in Mental Health Service Delivery in Ireland. *The Journal of Mental Health Training, Education and Practice*, 19(2), 96–107.
Narrative recovery	(1) Kerr, D. J., Crowe, T. P., and Oades, L. G. (2013). The Reconstruction of Narrative Identity During Mental Health Recovery: A Complex Adaptive Systems Perspective. *Psychiatric Rehabilitation Journal*, 36(2), 108. (2) Spector‐Mersel, G., and Knaifel, E. (2018). Narrative Research on Mental Health Recovery: Two Sister Paradigms. *Journal of Mental Health*, 27(4), 298–306. (3) Robertson, S. J., Carpenter, D., and Donovan‐Hall, M. (2017). ‘From the Edge of the Abyss to the Foot of the Rainbow–Narrating a Journey of Mental Health Recovery’ the process of a wounded researcher. *The Qualitative Report*, *22*(8). (4) Llewellyn‐Beardsley, J., Rennick‐Egglestone, S., Pollock, K., Ali, Y., Watson, E., Franklin, D.,… and Edgley, A. (2022). ‘Maybe I shouldn't talk’: The Role of Power in the Telling of Mental Health Recovery Stories. *Qualitative Health Research*, 32(12), 1828–1842. (5) Kaiser, B. N., Varma, S., Carpenter‐Song, E., Sareff, R., Rai, S., and Kohrt, B. A. (2020). Eliciting Recovery Narratives in Global Mental Health: Benefits and Potential Harms in Service User Participation. *Psychiatric Rehabilitation Journal*, 43(2), 11. (6) Hui, A., Rennick‐Egglestone, S., Franklin, D., Walcott, R., Llewellyn‐Beardsley, J., Ng, F.,… and Slade, M. (2021). Institutional Injustice: Implications for System Transformation Emerging From the Mental Health Recovery Narratives of People Experiencing Marginalisation. *Plos One*, 16(4), e0250367. (7) Rennick‐Egglestone, S., Ramsay, A., McGranahan, R., Llewellyn‐Beardsley, J., Hui, A., Pollock, K.,… and Slade, M. (2019). The Impact of Mental Health Recovery Narratives on Recipients Experiencing Mental Health Problems: Qualitative Analysis and Change Model. *PloS One*, 14(12), e0226201.
Social recovery	(1) Best, D., Beckwith, M., Haslam, C., Alexander Haslam, S., Jetten, J., Mawson, E., and Lubman, D. I. (2016). Overcoming Alcohol and Other Drug Addiction as a Process of Social Identity Transition: The Social Identity Model of Recovery (SIMOR). *Addiction Research & Theory*, 24(2), 111–123. (2) Marino, C. K. (2015). To Belong, Contribute, and Hope: First Stage Development of a Measure of Social Recovery. *Journal of Mental Health*, 24(2), 68–72. (3) Mezzina, R., Davidson, L., Borg, M., Marin, I., Topor, A., and Sells, D. (2006). The Social Nature of Recovery: Discussion and Implications for Practice. *Archives of Andrology*, 9(1), 63–80. (4) Topor, A., Borg, M., Di Girolamo, S., and Davidson, L. (2011). Not Just an Individual Journey: Social Aspects of Recovery. *International Journal of Social Psychiatry*, 57(1), 90–99. (5) Pahwa, R., Smith, M. E., Yuan, Y., and Padgett, D. (2019). The Ties That Bind and Unbound Ties: Experiences of Formerly Homeless Individuals in Recovery From Serious Mental Illness and Substance Use. *Qualitative Health Research*, 29(9), 1313–1323. (6) Topor, A., Borg, M., Mezzina, R., Sells, D., Marin, I., and Davidson, L. (2006). Others: The Role of Family, Friends, and Professionals in the Recovery Process. *Archives of Andrology*, 9(1), 17–37. (7) Schön, U. K., Denhov, A., and Topor, A. (2009). Social Relationships as a Decisive Factor in Recovering From Severe Mental Illness. *International Journal of Social Psychiatry*, 55(4), 336–347. (8) Skogens, L., von Greiff, N., and Topor, A. (2018). Initiating and Maintaining a Recovery Process–Experiences of Persons With Dual Diagnosis. *Advances in Dual Diagnosis*, 11(3), 101–113. (9) Tew, J., Ramon, S., Slade, M., Bird, V., Melton, J., and Le Boutillier, C. (2012). Social Factors and Recovery From Mental Health Difficulties: A Review of the Evidence. *British Journal of Social Work*, 42(3), 443–460. (10) Vera San Juan, N., Gronholm, P. C., Heslin, M., Lawrence, V., Bain, M., Okuma, A., and Evans‐Lacko, S. (2021). Recovery From Severe Mental Health Problems: A Systematic Review of Service User and Informal Caregiver Perspectives. *Frontiers in Psychiatry*, 12, 712026. (11) Damsgaard, J. B., and Angel, S. (2021). Living a Meaningful Life While Struggling With Mental Health: Challenging Aspects Regarding Personal Recovery Encountered in the Mental Health System. *International Journal of Environmental Research and Public Health*, 18(5), 2708. (12) Ramon, S. (2018). The Place of Social Recovery in Mental Health and Related Services. *International Journal of Environmental Research and Public Health*, 15(6), 1052.
Family recovery	(1) EnglandKennedy, E. S., and Horton, S. (2011). “Everything That I Thought That They Would Be, They Weren't:” Family Systems as Support and Impediment to Recovery. *Social Science & Medicine*, 73(8), 1222–1229. (2) Glynn, S. M., Cohen, A. N., Dixon, L. B., and Niv, N. (2006). The Potential Impact of the Recovery Movement on Family Interventions for Schizophrenia: Opportunities and Obstacles. *Schizophrenia Bulletin*, 32(3), 451–463. (3) Wyder, M., and Bland, R. (2014). The Recovery Framework as a Way of Understanding Families' Responses to Mental Illness: Balancing Different Needs and Recovery Journeys. *Australian Social Work*, 67(2), 179–196. (4) Bland, R., and Wyder, M. (2024). Families and Recovery: Beyond Clinical and Social Inclusion Perspectives. *Social Work and Social Sciences Review*, 25(1), 137–146. (5) Reynolds, D., McMahon, A., and McMahon, J. (2022). Being Held Through Pain: An Interpretative Phenomenological Analysis of Experiences of Receiving a Peer Support Intervention for Family Members of Individuals With Mental Illness. *Counselling and Psychotherapy Research*, 22(3), 736–747.
Cultural diversity in recovery	(1) Adeponle, A., Whitley, R., and Kirmayer, L. J. (2012). Cultural Contexts and Constructions of Recovery. *Recovery of People With Mental Illness: Philosophical and Related Perspectives*, 109–132. (2) Jacobson, N., and Farah, D. (2012). Recovery Through the Lens of Cultural Diversity. *Psychiatric Rehabilitation Journal*, 35(4), 333. (3) Bryant‐Davis, T. (2019). The Cultural Context of Trauma Recovery: Considering the Posttraumatic Stress Disorder Practice Guideline and Intersectionality. *Psychotherapy*, 56(3), 400.
Relational recovery	(1) Brekke, E., Ness, O., and Lien, L. (2020). Relational Recovery in Co‐Occurring Conditions: A Qualitative Study of First‐Person Experiences. *Advances in Dual Diagnosis*, 13(2), 89–100. (2) Price‐Robertson, R., Obradovic, A., and Morgan, B. (2017). Relational Recovery: Beyond Individualism in the Recovery Approach. *Advances in Mental Health,* 15(2), 108–120. (3) Korsbek, L. (2016). Corecovery: Mental Health Recovery in a Dynamic Interplay Between Humans in a Relationship. *American Journal of Psychiatric Rehabilitation*, 19(3), 196–205. (4) Bjørlykhaug, K. I., Bank, R. M., and Karlsson, B. E. (2020). Social Support and Relational Recovery in the Age of Individualism: A Qualitative Study Exploring Barriers and Possibilities for Social Support in Mental Health Work. (5) Schön, U. K., and Rosenberg, D. (2013). Transplanting Recovery: Research and Practice in the Nordic Countries. *Journal of Mental Health*, 22(6), 563–569.

## 3 Results

The selected articles allow researchers to reconstruct the plurality of theoretical and analytical lenses through which the construct of recovery has been interpreted over time. Table 3 shows the definitions of each recovery construct that emerged from the in‐depth narrative analysis of the articles. As Green (2006) suggests, a narrative review is a comprehensive narrative synthesis of previously published information, aimed at presenting a broad perspective on a topic, describing its history or development, useful for provoking reflection and controversy. Subsequently, we describe for each recovery construct the resources and limitations, highlighting ambiguities and operational difficulties in the application of the constructs, or even the elements that can reveal the epistemological assumptions to which the papers refer.

**Table 3:** Definitions of recovery constructs.

The ‘Clinical Recovery’ or ‘Recovery’	The term ‘clinical recovery’ refers primarily to the reduction of psychiatric symptoms through a curative approach to the illness using medications and psychotherapy [23]. This conceptualisation, which Davidson & Roe [10] define as ‘recovery from’, derives from longitudinal studies regarding the outcomes of severe mental illness; it assumes that recovery (partial or complete recovery) is not only possible, ‘but is at least as common an outcome in severe mental illness, if not more so, than in severe and persistent impairment’ ([10], p. 462).
The personal recovery or ‘Recovery in’	The shift to a conceptualisation of recovery that focuses not on the illness and the symptom, but rather on the person in their own life system and its meanings, is also described as ‘recovery in’ [10]. This definition originates in the independent living and civil rights movement of survivors in the 1960s and 1970s [10]. Here, recovery is subjectively defined by the person to the extent that the individual feels he or she has regained some control over his or her life. ‘It is when people with mental illnesses are most disabled by the illness that their human and civil rights and responsibilities become most pressing and relevant. In a Recovery from versus recovery in serious mental illness analogous fashion to physical disability, recovery in mental illness speaks primarily to the person's rights of social inclusion and self‐determination despite the severity of his or her psychiatric condition’ ([10], pp. 465–466).
The narrative recovery	Within the personal recovery perspective, we can trace a further qualification of the construct arising from the adoption of different theoretical perspectives, particularly the constructivist and narrative paradigms [24]. Research discussing ‘recovery narratives’ [24, 25] supports the therapeutic use of storytelling [26, 27] and defines ‘recovery narratives’ as the stories of psychological distress from which survival, agency and recovery emerge for people who have experienced it themselves [24]. In this sense, storytelling is not only a tool for recovery, but the ability to tell one's story, i.e. to take control of it, becomes evidence of one's recovery [28].
The social recovery	As highlighted in the literature on personal recovery, recovery takes place thanks to the person's efforts, his or her ability to acquire a greater sense of hope and empowerment [13]; however, for other authors, recovery is not to be understood as a personal and unique process for each individual, but rather as a pathway that takes place within and thanks to a social and interpersonal context [29, 30, 31]. As Hopper [32] considers, within a social conception, there is an insistence on the idea that the ‘mental health programme rejects therapeutic individualism in favor of understanding persons as social beings embedded in networks of distinction and entitlement that reproduce broader material inequities and ratify rank orders of regard’ (p. 877). This definition of recovery is strongly asserted by service users and caregivers. This is what the systematic review of [33] highlights: service users' main understanding of recovery emphasises connection with others and the ability to exercise agency and control over one's own problems and the achievement of economic self‐sufficiency.
The family recovery	Compared to social recovery, we could say that a ‘narrowing of the social field’ takes place. As Wyder and Bland [34] argue, mental illness emerges not only within broader social processes such as culture, economics and education but also in the context of the individual's network of family and personal relationships. Indeed, recovery from mental illness does not occur in isolation but also through the support of one's partner, friends or others. These play an active role in assisting the person with mental illness, in maintaining confidence, in supporting a new view of oneself and in encouraging one's autonomy.
Cultural diversity in recovery	The assumptions of personal recovery have also been challenged by those works that adopt the cultural and contextual dimensions of the recovery process as pre‐eminent. Indeed, these works highlight that current notions of recovery are based on the concept of the individualistic or egocentric person [35], as well as on the Euro‐American assumptions of neoliberalism, which emphasise the values of independence, autonomy and personal fulfilment [2]. The predominance of Euro‐American definitions in the literature on recovery has raised questions about the applicability of the construct in other cultures: ‘are current notions of recovery suitable for dealing with the experience of other peoples and cultures? How are illness trajectories, adjustment patterns, preference and response to common recovery interventions composed elsewhere? What does “successful outcome” mean elsewhere?’ ([2], p. 122). If recovery goals are grounded in Western cultural values, beliefs and norms, they are unsuitable or perhaps inapplicable in other contexts [36], as shown by Tse and Ng [37].
The relational recovery	Relational recovery is based on post‐Cartesian psychological theories—in their systemic, ecological, intersubjective and relational forms, which assume the constitutively relational nature of the human being [38, 39, 40]. Relational recovery stands in continuity with conceptualisations of social and family recovery, while differing from them, and contrasts with the individualistic drifts of personal recovery, as it considers that the latter risks being overly focused on people's internal and subjective experiences, undervaluing the interpersonal contexts of recovery [41, 42]. This perspective is revolutionising the way recovery is conceptualised insofar as it reformulates the crucial elements of recovery, such as hope and healing, as relational concepts, and suggests expanding the investigation of recovery by investigating what is breaking community and relations [43].

### 3.1 Resources and Limitations for Each Recovery Construct, Available Ambiguities and Operational Difficulties in Its Application

#### 3.1.1 The ‘Clinical Recovery’

Clinical recovery views mental illness as an extension of organic illness. According to Davidson, this conception has many advantages from both a clinical and research perspective, being ‘clear, reliable and relatively easy to define, measure and link to dysfunction or well‐being in other areas of life’ ([10], p. 463). However, it is precisely the narratives of people in recovery that suggest that the partial or total disappearance of symptoms, or even the mere reading of mental illness in symptomatic terms, is a very limiting assumption. One cannot ignore the enormous work that needs to be done to ‘recover from the social and personal impact of the illness’ ([10], p. 463).

#### 3.1.2 The ‘Personal Recovery’

The focus on the person as a rights holder and as an agent responsible for his or her health has enabled the initiation of collaborative research designs, where participants have taken on the role of co‐researchers [44, 45, 46, 47]. Elements such as hope, expectations, self‐esteem, self‐efficacy, self‐awareness, self‐determination, responsibility and personal dignity are considered ‘crucial ingredients of subjectivity in recovery’ ([48], p. 2). Despite the invitation to pay more attention to the subjectivity of people in recovery, research has found a lack of theoretical clarity on the task of supporting personal recovery in service practice, where the definition of recovery offered by staff does not always align with that proposed by users [49]. Stuart et al. [50] and Van Weeghel et al. [51] highlight the need to broaden the conceptualisation of personal recovery by taking into consideration the difficulties that people who embark on these paths may encounter. Celebrating only the strengths of those who appear to be successful might perpetuate the idea that recovery is something attainable by all who simply apply themselves. This carries with it the risk of blaming and stigmatising as ‘those who do not try hard enough’ people who struggle with the concepts of healing ([5] in [50]). The overwhelming pressure to pursue ‘successful’ paths to recovery is closely shaped by the neoliberal logic that subsumes recovery as a performance activity based on individualism and personal empowerment [52].

#### 3.1.3 The ‘Narrative Recovery’

Narrative reconstruction of one's own recovery story can benefit the storyteller [53]. Listening to other's stories can be helpful for those who have experienced similar mental distress; sharing stories provides listeners with validation, empowerment, hope and alternative conceptualisations of their suffering [27]. Peer support is based on building this kind of relational connection. However, several studies show that the storytelling of recovery may involve power dynamics. Llewellyn‐Beardsley et al. [54] provide a comprehensive overview of how power dynamics influence the ways lived experience stories are told. Stories of mental health recovery are often shaped and constrained by dominant cultural narratives and definitions bestowed by services: these norms guide the narrative and select what can and cannot be said. Unacceptable narratives include aspects such as anger, experiences of abuse, political engagement and epistemic oppression [54, 55], as well as those narratives that use language that does not match that of the institutions they address [56, 57].

#### 3.1.4 The ‘Social Recovery’

Two strands of research emerged by using the label ‘social recovery’. The first emphasises social relationships, both with family and friends, but also with professionals and other mental health service users, as decisive for recovery from serious mental illness [58, 59]. Subsequent studies have questioned the dynamics through which relationships may promote the processes of change. Best et al. [20] argue that the feeling of belonging to a recovery‐oriented group, with its norms and values, may promote processes of change through new self‐categorisations that emancipate the person from his/her problematic past and by the acquisition of a new positive identity. Hence the importance of creating an accepting and favourable social environment to support recovery [60, 61]. In this first strand, inspired by concepts and models from social psychology, a ‘dualistic’ view of the person in recovery persists [62]. There is a second strand in which authors refer to the ‘social’ using concepts such as ‘community’ and ‘citizenship’, in continuity with the vision of recovery claimed by the ex‐patients movement, which focuses very much on the social inclusion of people and their citizenship rights [63, 64]. For Mezzina [63, 64], recovery is an interactive journey [64] that includes the occupation of valued roles, the exercise of personal authority, the development of a positive identity and participation in the community [63]. This understanding of recovery implies a new way of understanding mental health service users: the person is seen as a free citizen who expresses agency on a personal, social, political and cultural level [64]. Not only is he or she able to indicate to the service the strategies of his or her own recovery, but, as an expert on the services, he or she is also qualified to propose indications for improving their organisation [64].

#### 3.1.5 The ‘Family Recovery’

Family recovery emphasises the importance of caregivers in recovery processes. Health professionals have to be able to support caring relationships, offering support to the family, including it in therapeutic processes and offering psycho‐educational interventions [34, 36, 65]. Family members often support recovery through ‘intangible aids’.

Also, clients report that the main support is derived from their family, and, in particular, from the feeling of being accepted by their family [65, 66]. Social work practice with families is an element that can improve the recovery process [34]. The open dialogue approach, in continuity with systemic therapy, sees the person who manifests symptoms only as the designated patient within the system, the one who bears the symptomatic burden. The approach considers that work on family communication must be introduced as a prerequisite for keeping dialogue alive and active in all relationships [67]. The healing process regards the entire family [68] as performed by the Family Recovery Peer Support programmes [69].

### 3.2 A Cultural Perspective on Recovery

An understanding of the cultural dimensions of recovery requires attention to two levels: on one hand, to the discursive level, where social policies shape the theory and practice of recovery, and on the other hand, to the lived experience, that is, how the values and ways of life of ethnocultural communities shape the concept of recovery [2]. The universalisation of the concept of recovery can lead to the invisibilisation of the social, structural, political and economic dimensions that produce suffering [2]. Instead, a cultural focus leads the clinician to consider the sociocultural realities that permeate the internal and external world of the person in recovery [70]. For example, a clinician working with people from marginalised communities cannot fail to consider that they may have experienced genocide, incarceration, poverty, systemic discrimination and colonialism (Hartmann et al., 2019 in [70]). This perspective foregrounds culture, systems of oppression and privilege, social determinants of health and history, arguing that these factors are central to healing ([71]; Skewes and Blume, 2019 in [70]). Such intersectional attention can allow the complexity of people's lives to be respected [70].

### 3.3 The ‘Relational Recovery’

The conceptualisation of ‘relational recovery’ aims to overcome the Cartesian dualism underlying such a dichotomy between individual and context, thus between internal and external factors. Identity does not belong to the individual but is composed of the dialectic between the individual and society. The role does not exist apart from the fact that someone recognises and confirms it: this is the basis for the co‐construction of personal identity. These premises have given rise to the idea that recovery should be co‐constructed in the relationship between users and practitioners, recognising that both parties have vital contributions to make [72], hence the increasing emphasis on co‐production and co‐creation experiences and emerging conceptions in the field of mental health [73]. Many authors, ignoring these roots, use the adjective ‘relational recovery’ to support arguments relevant to ‘social recovery’ [60, 74]. The quality of social support increases when patients are offered stable meeting places where they can support each other, find comfort and reflect together on dynamics such as feelings of stigmatisation [43]. However, practitioners must work to ensure inclusion and collaboration with service users to create the conditions for their experiences to challenge the diagnostic culture, which is still strongly present in services [43]. In this respect, the concept of relational recovery has also inspired new perspectives, including that of *natural recovery*. ‘The processes of *natural recovery* are seen as specific relational trajectories or transformative pathways involving relationships between humans, non‐humans, communities, and philosophies, rather than as a process of symptom elimination. This kind of conceptualization of recovery acknowledges the many people who manage to recover without treatment or professional help’ ([75], p. 1).

## 4 Discussion

Various configurations of recovery emerged from our analysis; one wonders how similar they are and how they differ. The construct of clinical recovery is the oldest, linked to the medical tradition. It maintains the focus on the body, on the organic root to which the symptomatology is attributed and on the pharmacological therapy considered to correspond. However, there is a developing literature problematising the overlap between the disappearance of the symptoms and the quality of healing [76]. This research has brought into focus the value of how people interpret and give personal meaning to the recovery experience. A striking example of the disastrous effects of the generalisation and dominance of the medical model of recovery, at the expense of the many ways in which users can construct a wholly personal representation of recovery, comes from research conducted by the University of Manchester in 2014 [14]. With a substantial number of discharges from mental health services, the outcome of a favourable recovery assessment, over the 10 years 2003–2013, as many as 2368 patients committed suicide within the first 3 months of discharge, with a peak in the first week. All participants in the study stated that they felt ready to return to normal life. Their level of recovery was assessed according to the instruments adopted by the service, that is, according to biomedical criteria internalised by the users themselves during their rehabilitation.

This example testifies to the urgency of overcoming the idea that a certain model of recovery, insofar as it is institutionalised, is representative of all forms of recovery while emphasising the importance of diversifying recovery constructs as much as possible, and above all allowing users to create their own, personal representation of what are their fundamental needs and requirements, indicative of an improvement on a life plan of personal, existential, relational and community meaning and not only symptomatological [77]. The concept of personal recovery has emerged right from the consumer movement in the past 20 years as a reaction to the highlighted limitations.

Here, personal recovery has several characteristics that focus on outcomes that are important to the individual and distinguish it from more clinically based models: it is individually defined through an understanding of the narrative, and it is based on the concept of journey, growth and development. It focuses primarily on social success and individually defined forms of progress, rather than on symptom control. Recent research highlights how clinical and personal recovery does not mix and match: the first seems to be much more closely related to the quality of life of the users rather than to symptom remission [78]. It started from a reversal of perspective concerning the sources of knowledge: to understand what recovery means, it is not useful to start from medical diagnostics, but from people's life experience [79, 80, 81]. Thus began the research on life stories: the power to define reality passed from the hands of the physician to the hands of the user. Hence the construct of ‘recovery narratives’. The use of recovery narratives increased rapidly within services, and the value of testimony became crucial to instil confidence and hope for recovery; the so‐called ‘Ebe’ (Experts by Experience) was introduced as a resource for services in almost every way [82, 83]. They represent, in some ways, a challenge to the medical model, as they emphasise the relational, social and environmental factors that contribute to mental distress [84]. By using their life experiences to provide non‐medical support, they counter the medicalization of mental illness and seek to reduce the stigma associated with psychiatric labels [85].

With them, interest in investigating the ‘ingredients of recovery’ across stories has also increased. Recovery narratives are diverse and multidimensional. They can be nonlinear and reject coherence [26]. To a greater extent than illness narratives, they incorporate social, political and rights aspects. Approaches to support the development of healing narratives should broaden rather than reduce the choices available. Research on the narratives of more diverse populations is therefore needed.

One can well understand how this was only the start of the proliferation of the other recovery constructs we have considered: hence, in addition to personal recovery, cultural recovery, all of which bear a greater tolerance of diversity and the need to complexify and relativise knowledge and theoretical models on recovery.

Another important consideration should be made regarding those recovery constructs that focus on social and relational aspects. Modern approaches consider the person to be the fundamental unit of the identity process, and that the individual experience is constructed along a contrast between internal and external factors; these approaches to which social recovery and also personal recovery can be referred consider relationships as external factors.

They are highly relevant but successive and separable from personal identity; conversely, the construct of relational recovery and that of cultural recovery consider the individual intrinsically relational and culturally/linguistically generated. Overcoming the dichotomy between internal and external factors, typical of the Cartesian model, that hold an atomic view of individuality, thought, and responsibility for action, they embrace a view of identity much more contextual and linked to the discourses and meanings expressed in interaction. Thoughts are understood here not as property of the individual but as the effect of the dialogue with both others and oneself. A different, anti‐realist and anti‐objectivist epistemology comes into play, as does a different representation of personal identity. Adopting this position may allow the practitioner to broaden how recovery is understood. Rather than anchoring it solely in the remission of symptoms, it may broaden the meaning that recovery has for the patient.

A further important differentiation must be made concerning the systemic approach and family recovery, an approach in which the individual disappears and the basic unit of the process that generates distress or heals becomes the whole family system. Here, the whole family system is considered a ‘patient in care’ since psychosis is seen as happening between people, not within a person. Focusing on helping improve the social relationships surrounding the person in crisis is the key to recovery.

The problems are addressed in the network of relationships surrounding a person who is ‘in crisis’, rather than assuming the problem is inside the person's head [67]. Helping the social network—including changing attitudes of providers—rather than just focusing on achieving change in the person in crisis. The problem is seen between people, and in the broader social context, it is not considered a pathology in the individual. This type of interpretation of discomfort, which is contextual and relative to the whole system, implies different methods and tools for working on relationships and requires other ways of assessing recovery in terms of the system's openness and maintenance of dialogue.

One understands, therefore, that talking about ‘relational competence’ or ‘relationships’ is not enough; one needs to identify from which theoretical construct that certain way of understanding relationships and the individual was conceived.

## 5 Conclusion

The ‘heterogeneity of perspectives’ and lenses through which recovery is investigated reflects the complexity of the interpretation of mental distress and the presence of different cognitive paradigms, still coexisting in the literature, whose level of interconnection has not yet been clarified or even resolved. Mental health is not a self‐evident, self‐proclaiming reality; it can only be investigated through a series of constructs (the result, as the term itself implies, of linguistic constructions learned and negotiated in discourse) that derive from having carved out phenomena according to a certain language, a language at the basis of which there are many strongly cultural aspects, of which individuals forget, mistaking mental distress and the path to recovery for factual reality. Hence, a need for greater scientific rigour and more accurate awareness on the part of researchers and practitioners.

Such an awareness translates:

(1) In the disillusionment of referring to the same phenomenon where the same term (‘recovery’) is used without the clarification of the reference theory, the ‘unconscious’ use of the term risks, in fact, to make true any kind of action defined as recovery‐oriented (as it happens for the term ‘therapy’) [86, 87, 88] without questioning the theoretical principle and therefore the conditions within which its effectiveness is expressed;

(2) From the point of view of clinical operation, this makes it inadmissible to use instruments conceived within a different theoretical frame to evaluate only apparently related constructs misleadingly and confusingly.

It is not enough to be satisfied with the name (e.g., social recovery); one needs to understand how that construct is interpreted and which tools are in line with the theoretical definition of the construct in question. On the contrary, the specialist literature often neglects the construct definitions and imagines ‘saying’ or ‘evaluating’ recovery as if it were a real object rather than a metaphor, which can be meant in various and multiple ways.

(3) Another important call from our review is to provide mental health workers with more knowledge of the recovery constructs available in the literature and to broaden the range of discourses and interpretative theories to share with users, to accompany them in the process of personally constructing a representation of their recovery journey and its differences from the stories of others. This would allow them to be more attentive and avoid influencing or overwriting their model, the user's vision of themselves and their own healing needs.

(4) It should also be considered that our review highlights the emergence of critical studies that show how the neoliberal agenda, by emphasising individual responsibility, has obscured the crucial role of social factors and material and cultural change in recovery. In this regard, Topor [89] proposes an updated perspective of the well‐known definition of recovery proposed by Anthony, aimed at paying attention to these aspects.

− ‘Recovery is a deeply social, unique, and shared process in which our living conditions, material surroundings, social relations and sense of self evolve.
− It is about striving to live satisfying, hopeful, and reciprocal lives, even though we may still experience threats, stressful social situations, and distress.
− Recovery involves engaging in encounters and dialogs where new ways of understanding and handling one's situation are created as we move beyond the psycho‐social‐material crisis.’ ([89], p. 11).

Recognising the interaction between the personal dimension of recovery and its relational, cultural and material aspects allows for a deeper understanding of the phenomenon and offers mental health professionals a more effective perspective for their intervention.

Our review has focused on the risk of generalising and the unquestioned tendency to reify, in terms of content, an experience that cannot be defined as such, if not based on shared and always fluctuating meanings. Indeed, it should be remembered that the construct of recovery does not indicate a permanent state or mode of being but a complex and changing experience of self that results from mixing one's present with the past, the here and now with the future. The resulting feeling towards oneself is iridescent and closely linked to the context and circumstances within which the person lives. An interesting and stimulating research topic to be explored would be that of investigating how the idea of recovery best suited to oneself changes longitudinally in the various phases of the pathway, before and after contact with the services, by virtue of the contamination with other histories, or even in correspondence with the negotiation of meanings relating to one's history that takes place in dialogue with mental health professionals.

### 5.1 Relevance to Clinical Practice

Keeping theoretical descriptions and models of healing open and plural means enabling mental health practitioners not to monologise discourses of change by imposing their point of view on users. It means supporting users to authentically seek their healing pathways without having to conform to clinicians' expectations, abandoning misleading and naive simplifications and strictly using the appropriate terms relevant to the specific healing construct that researchers refer to from time to time.

## Author Contributions

All authors contributed to the study's conception and design. Material preparation, literature data collection, analysis and article drafting were performed by Elena Faccio, Ludovica Aquili, Michele Rocelli, Lia Bitetti, Susanna Brunelli and Federica Mangione. Giuseppe Salamina revised it critically for important intellectual content. All authors read and approved the final manuscript. Co‐first/equal authorship: Elena Faccio and Ludovica Aquili provided equal contributions.

## Ethics Statement

The authors have nothing to report.

## Consent

The two peer support workers (ex‐patients) who comprise the research team, contributed equally to each step of the research, including conceptualisation, methodology of research, results, and the relevance of results publication.

## Conflicts of Interest

The authors declare no conflicts of interest.

## Data Availability

The authors have nothing to report.
